# Characterisation of Pain Responses in the High Fat Diet/Streptozotocin Model of Diabetes and the Analgesic Effects of Antidiabetic Treatments

**DOI:** 10.1155/2015/752481

**Published:** 2015-02-09

**Authors:** Frederika Maria Byrne, Sharon Cheetham, Steven Vickers, Victoria Chapman

**Affiliations:** ^1^School of Life Sciences, University of Nottingham, Nottingham NG7 2UH, UK; ^2^RenaSci Ltd., BioCity Nottingham, Pennyfoot Street, Nottingham NG1 1GF, UK; ^3^Arthritis Research UK Pain Centre, University of Nottingham, Nottingham NG7 2UH, UK

## Abstract

Chronic pain is a common complication of diabetes. The aim of the present study was to characterise pain behaviour in a high fat diet/streptozotocin (HFD/STZ) model of diabetes in the rat, investigate spinal mechanisms, and determine the effects of antidiabetic interventions. Three-week consumption of a high fat diet followed by single injection of STZ (45 mgkg^−1^) produced sustained changes in plasma insulin and glucose until day 120. Hindpaw mechanical withdrawal thresholds were significantly lowered in the model, but mechanically evoked responses of spinal neurones were unaltered, compared to HFD/vehicle rats. HFD/STZ rats had significantly lower numbers of spinal Iba-1 positive cells (morphologically identified as activated microglia) and spinal GFAP immunofluorescence (a marker of astrogliosis) in the spinal cord at day 50, compared to time-matched controls. The PPAR*γ* ligand pioglitazone (10 mgkg^−1^) did not alter HFD/STZ induced metabolic changes or hindpaw withdrawal thresholds of HFD/STZ rats. Daily linagliptin (3 mgkg^−1^) and metformin (200 mgkg^−1^) from day 4 after model induction did not alter plasma glucose or insulin in HFD/STZ rats but significantly prevented changes in the mechanical withdrawal thresholds. The demonstration that currently prescribed antidiabetic drugs prevent aberrant pain behaviour supports the use of this model to investigate pain mechanisms associated with diabetes.

## 1. Introduction

It is estimated that 285 million people are currently living with diabetes, and it has been predicted that this will grow to 366 million by 2030 [1], with 90% having type 2 diabetes [2]. Neuropathy occurs in 50–60% of patients [3] and pain manifests in around 30% of diabetic patients with neuropathy [4]; symptoms include tingling, burning, spontaneous pain, allodynia, and hyperalgesia.

Preclinical investigations of the mechanisms underlying diabetic neuropathy have primarily used the administration of streptozotocin (STZ) in rodents to selectively destroy pancreatic *β*-cells [5], which models aspects of type 1 diabetes. This model of diabetes exhibits altered pain responses, including a lowering of hindpaw withdrawal thresholds, increased spontaneous activity of spinal neurones, and alterations in evoked responses of neurones [6, 7], all of which are indicative of the presence of central sensitization [8]. An increase in the numbers of activated spinal microglia [9, 10] and a decrease in a marker of spinal astrogliosis [11, 12] have been reported in STZ rats.

Developments in the modelling of diabetes have included the investigation of how diet may influence the progression and profile of diabetic neuropathy through the incorporation of a high fat diet (HFD) prior to treatment with a lower dose of STZ [13–15]. HFD is associated with hyperinsulinemia in mice [15], and exposure to the low dose STZ treatment destroys a proportion of the *β*-cells leading to a decrease in insulin production and the development of overt hyperglycaemia [13, 15–18]. The utility of this model to investigate pain mechanisms associated with diabetes is unclear, in the mouse both HFD alone and HFD/STZ produced similar increases in pain response frequency to a fixed mechanical stimulus, with only subtle differences in the latency of withdrawal to this stimulus significant between the nondiabetic HFD group and the HFD/STZ group [15]. By contrast in the rat, HFD was not associated with changes in mechanical or thermal pain behaviour, but the combination with STZ increased pain response frequency to a fixed mechanical stimulus whilst having inconsistent effects on mechanical thresholds [18, 19]. Given that some of the inconsistencies in the outcomes of these previous studies may reflect the limited period over which pain behaviour was assessed, further investigation of this model over a longer timeframe is warranted. The aim of the current study was to further investigate the potential translational utility of the HFD/STZ model of diabetes in the rat by quantifying changes in thresholds for mechanical withdrawal responses, akin to pain pressure thresholds in humans. To consolidate the evidence that changes in mechanical withdrawal thresholds are specific to diabetes, the effects of chronic administration of clinically effective antidiabetic drugs on this measure of pain response in the HFD/STZ rat were determined. Specifically, effects of the DPP-4 inhibitor, linagliptin, the insulin sensitising agent, metformin, and the nuclear receptor peroxisome proliferator-activated receptor gamma (PPAR*γ*) agonist, pioglitazone [20–22] on mechanical withdrawal thresholds in HFD/STZ versus HFD/Veh groups, were evaluated. The potential spinal neuronal and glial cell responses associated with HFD/STZ pain behaviour were also investigated to determine their potential contribution to the aberrant pain behaviour exhibited in this model.

## 2. Methods

### 2.1. Experimental Animals

All experiments were carried out in accordance with the UK Home Office Animals (Scientific Procedures) Act 1986 and data are reported in line with the ARRIVE guidelines [23]. All data were collected in a blinded fashion. Male Sprague-Dawley rats (200–250 g), from Charles River (Margate, Kent), were individually housed on a normal light cycle (lights on: 07:00–19:00) with free access to water and a high fat diet (60% fat by caloric content; D12492 diet; Research Diets, New Jersey, USA) or normal chow. Food and water intake, and body weight were monitored twice weekly. After three-weeks consumption of the HFD or chow, rats received an intraperitoneal (i.p.) injection of STZ (45 mgkg^−1^) or vehicle (0.05 M citrate buffer pH 4.5). STZ (batch/lot number 019K1022) was purchased from Sigma Aldrich (Poole, UK) and dissolved in 0.05 M citrate buffer pH 4.5. Three out of 67 rats injected with STZ did not develop hyperglycaemia and were excluded from further analysis.

### 2.2. Behavioural Assessment of Mechanical Hindpaw Withdrawal Thresholds

Calibrated von Frey monofilaments (Semmes-Weinstein monofilaments of bending forces 1, 1.4, 2, 4, 6, 8, 10, and 15 g) were used to quantify mechanical hindpaw withdrawal thresholds as previously described [24]. Mechanical withdrawal thresholds of both hindpaws were quantified twice-weekly for up to 17 weeks after STZ injection.

### 2.3. Metabolic Monitoring

Plasma glucose and insulin were assayed on days −5, 22, 43, 72, and 134. Following a four-hour fast, 100 *μ*L of blood was collected from the lateral tail vein and plasma was separated by centrifugation (2,400 g for 5 min at 4°C). Glucose and insulin concentrations were measured using standard assays (Thermoelectron Infinity glucose reagent and Mercodia rat insulin ELISA, resp.). In a subgroup of the rats studied, an oral glucose tolerance test (OGTT) was performed at day 120 (HFD/Veh: *n* = 6, HFD/STZ: *n* = 12). Rats were deprived of food overnight, and the following day the lateral tail vein was cannulated and a baseline blood sample was collected, along with an aliquot of whole blood for HbA1c determination, which is used as a measure of average plasma glucose concentration over a period of time (direct enzymatic HbA1c assay) [25]. Rats received a glucose load (2 g/kg p.o.), and blood samples were collected at 15, 30, 45, 60, 120, and 180 minutes after glucose loading. Plasma glucose and insulin concentrations were measured using standard assays as described above.

### 2.4. Administration of Drugs

The effects of oral linagliptin (3 mgkg^−1^; a kind gift from Boehringer Ingelheim Pharmaceuticals, Ingelheim, Germany) and metformin (200 mgkg^−1^; Sigma Aldrich, Poole, UK) on the development of HFD/STZ induced changes in mechanical hindpaw withdrawal thresholds were quantified, with the drugs administered from day 4 until day 40 and compared to saline vehicle (HFD/Veh/saline: *n* = 8; HFD/STZ/saline: *n* = 8; HFD/STZ/linagliptin: *n* = 7; HFD/STZ/metformin: *n* = 6).

The effects of oral daily pioglitazone (10 mgkg^−1^, in 1% methylcellulose; Tocris Cookson, Bristol, UK) versus vehicle on the development of HFD/STZ induced changes in mechanical hindpaw withdrawal thresholds were quantified once diabetes was established, from day 21 to day 49 after STZ treatment (*n* = 10-11 per group).

The dose of 3 mgkg^−1^ of linagliptin was used as this dose has been reported to improve glucose control when given once daily in animal models of diabetes [26]. In addition this dose significantly increased plasma GLP-1 in diet-induced obese rats and mice [27, 28] and caused 67–80% DPP-4 inhibition [28]. The doses of 200 mgkg^−1^ of metformin and 10 mgkg^−1^ of pioglitazone were selected as these doses prevent the development of, or reverse established, pain hypersensitivity when given orally in rats [29–32].

### 2.5. Spinal Electrophysiology

The properties and responses of spinal neurones were characterised in a subgroup of rats using established methods [24]. For these studies, anaesthesia was induced with 3% isoflurane in 66% N_2_O/33% O_2_. A cannula was inserted into the trachea, and the rat was placed into the stereotaxic frame. A spinal laminectomy exposed segments L4 and L5 of the spinal cord. Anaesthesia was maintained at 1.5% isoflurane and core temperature was monitored and maintained at 37°C.

A glass coated tungsten microelectrode was used to record from wide dynamic range (WDR) neurones (between 500 and 1000 *μ*m from the surface of the spinal cord, corresponding to laminae V-VI). Action potentials were digitised and analysed using a CED micro-1401 interface and Spike 2 data acquisition software (Cambridge Electronic Design, UK). Neurones which had a receptive field over one or two toes of the hindpaw and responded to both low and high weight von Frey hair stimulations were identified. Two fine stimulating needles were inserted into the receptive field, and the responses of the neurone to transcutaneous-electrical stimulation were recorded. Responses to a train of 16 stimuli (0.5 Hz, 2 ms pulse width), set at three times the threshold for C-fibre evoked responses, were recorded and categorised as follows: A*β*-fibre evoked (0–20 ms poststimulus), A*δ*-fibre evoked (20–90 ms poststimulus), C-fibre evoked (90–300 ms poststimulus), and postdischarge responses (300–800 ms poststimulus). Responses of the neurone to mechanical punctate stimulation of the peripheral receptive field were then characterised using von Frey hairs of different bending forces (8, 10, 15, 26, and 60 g), which were applied in ascending order for periods of 10 seconds per hair. The mean evoked frequency of firing, over the 10-second stimulation period, was recorded. The noxious withdrawal threshold to mechanical punctate stimuli in conscious animals is 15 g [33], and on this basis the range of von Frey hairs used included both innocuous and noxious stimuli.

### 2.6. Quantification of Spinal Glial Cell Activation

A subgroup of rats were overdosed with sodium pentobarbital and transcardially perfused with 0.9% saline followed by 4% paraformaldehyde (PFA; Sigma, UK) in 0.1 M phosphate buffered saline (PBS). The lumbar spinal cord was removed and postfixed in 4% PFA for 48 hours and then stored in 30% sucrose in 0.1 M PBS/0.02% sodium azide solution at 4°C. Immunohistochemical staining was performed on 40 *μ*m free-floating cryosections of L3/L4/L5 spinal cord. Microglial cells were stained for Iba-1 [34] and astrocytes were stained for GFAP [35]. Sections were blocked for 1 hour in 0.1 M PBS containing 3% normal goat serum and 0.3% Triton X-100 at room temperature. Sections were then incubated at 4°C for 72 hours with rabbit *α*-Iba-1 (Wako, Japan) diluted 1 : 1000 in Trizma Triton X-100 buffered saline (TTBS). Five 10-minute washes in 0.1 M PBS were carried out, and then sections were incubated for 2 hours at room temperature with Alexafluor 488 conjugated goat *α*-rabbit secondary antibody (Molecular probes, Oregon) diluted 1 : 500 in TTBS, and then another five 10 minute washes were carried out. This process was repeated on the same sections, this time incubating at room temperature for 18 hours with mouse anti-GFAP (Thermo-Fisher, Leicestershire, UK) diluted 1 : 100 in TTBS, and the Alexafluor 568 conjugated goat *α*-mouse secondary antibody (Molecular probes, Oregon) diluted 1 : 500 in TTBS for 2 hours at room temperature. Sections were then mounted on gelatinised microscope slides, air-dried overnight at room temperature in the dark and coverslipped using Fluoromount (Sigma).

### 2.7. Quantification of Antibody Staining

6–8 individual spinal cord sections were analysed per rat, and the mean of the left and right half of the dorsal and ventral horn was calculated for each section. Images for Iba-1 immunostaining and GFAP immunofluorescence were captured using a 20x 0.4 NA objective lens on a Leica DMIRE2 fluorescence microscope, running Volocity 5.5 (PerkinElmer) equipped with a Hamamatsu Orca C4642-95 camera and analysed using established methods [36].

Images of Iba-1 immunostaining were acquired using a typical exposure time of 444 ms. Total numbers of activated microglia expressing Iba-1 were counted manually in both the left and right sides of individual sections. Microglia were defined as activated if they displayed a clearly swollen cell body with reduced processes, which differ from normal or “resting” microglia where cell bodies are largely absent and large ramified processes are displayed. All counts were independently verified by a second experimenter. Background corrected mean fluorescence grey intensity was also quantified using IMAGE J (NIH open software with Macbiophotonics plugins).

For quantification of GFAP, single-plane images of the spinal cord were acquired on the predescribed system using an identical exposure time of 393 ms. Background corrected mean fluorescence grey intensity was also quantified using IMAGE J. All image analysis, cell counts, and fluorescence measurements were performed offline on captured images taken from stained sections.

### 2.8. Statistical Analysis

Analysis of body weight and food and water intake was by analysis of covariance (ANCOVA) with Tukey's post hoc test, with the average value for days −7 to 0 as a covariate. Analysis of plasma glucose, plasma insulin, the OGTT, HbA1c, and mechanical withdrawal thresholds was by either a Mann Whitney test or a Kruskal Wallis test with Dunn's post hoc test. Further analysis of the OGTT was by a Friedman test with Dunn's post hoc test. Analysis of immunofluorescence used a 2–way analysis of variance (ANOVA) with Bonferroni post hoc test. Analysis of electrical thresholds and latencies of evoked neuronal responses and magnitudes of mechanically and electrically evoked neuronal responses used a Kruskal Wallis test with Dunn's post hoc test. In all analyses, a *P* < 0.05 was considered statistically significant.

## 3. Results

### 3.1. Metabolic Changes in the HFD/STZ Model of Diabetes

Prior to the start of the study the rats were stratified into two groups based on body weight and hindpaw mechanical withdrawal thresholds. Rats on the high fat diet had a steady increase in body weight (average 53 g per week). Following injection of STZ, rats exhibited a dip in body weight and thereafter exhibited reduced body weight gain. By day 120, body weight was significantly (*P* < 0.001) higher in the HFD/Veh group compared to the HFD/STZ rats (792 ± 23 g versus 532 ± 17 g resp.: HFD/Veh: *n* = 6, HFD/STZ: *n* = 12). Water intake was significantly higher (*P* < 0.001) in the HFD/STZ group (88.3 ± 6.2 mL per day, compared to 22.0 ± 0.8 mL per day in the HFD/Veh group, at day 120).

**Figure 1:**
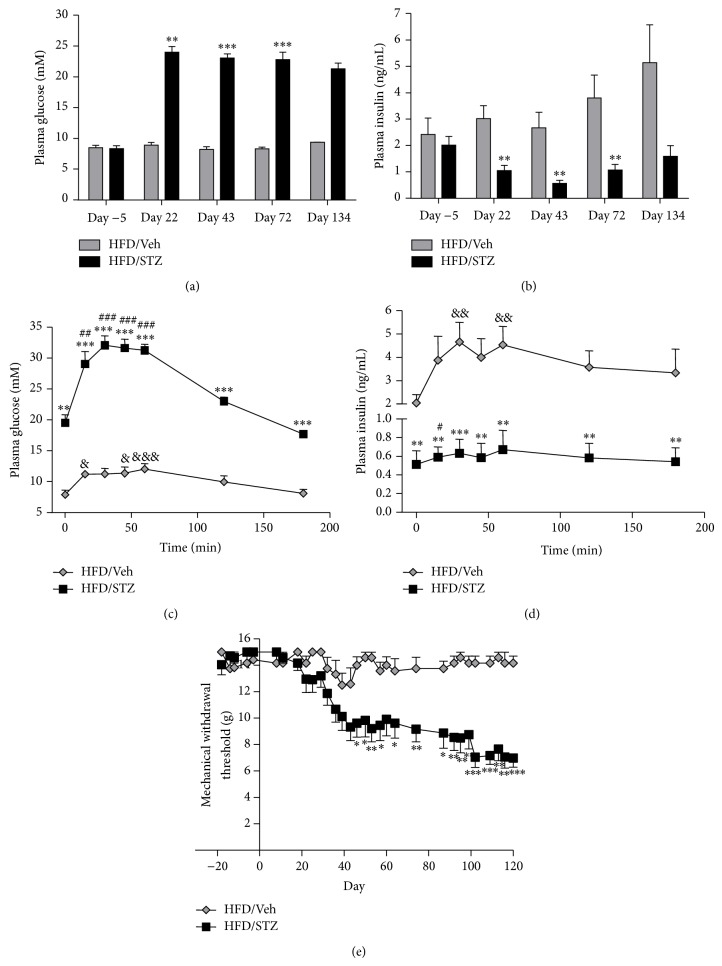
Effects of HFD/STZ on glucose and insulin levels, and mechanical withdrawal thresholds. Time course of the effects of HFD/STZ (*n* = 12 rats) on (a) fasting plasma glucose and (b) fasting plasma insulin, in comparison with HFD/Veh treatment (*n* = 6 rats). At day 120 an oral glucose tolerance test (OGTT) was performed and plasma glucose (c) and plasma insulin (d) were quantified in the two groups of rats. (e) Mechanical withdrawal threshold of the hindpaws was measured twice weekly for up to 120 days. Data are mean ± SEM. Comparisons between HFD/STZ and HFD/Veh rats were performed with a Mann-Whitney test: ^*^ *P* < 0.05, ^**^ *P* < 0.01, and ^***^ *P* < 0.001. For further analysis of the OGTT, comparisons to baseline values in each group were made using a Friedman test with Dunn's post hoc test: HFD/Veh ^&^ *P* < 0.05, ^&&^ *P* < 0.01, and ^&&&^ *P* < 0.001; HFD/STZ ^#^ *P* < 0.05, ^##^ *P* < 0.01, and ^###^ *P* < 0.001.

Three weeks after STZ administration, HFD/STZ rats had decreased plasma insulin and increased plasma glucose (Figures 1(a) and 1(b)). These robust metabolic changes were maintained until completion of the study (day 134 after STZ administration). To confirm impaired glucose control in STZ rats an oral glucose tolerance test (OGTT) was performed. Following the glucose load there was a significant (*P* < 0.01) increase in insulin secretion in the HFD/Veh group from 20–60 minutes, which returned to basal levels after 120 minutes (Figure 1(d)). This was associated with a small but significant (*P* < 0.05) increase in plasma glucose levels from 15 to 60 minutes (Figure 1(c)). By contrast, in the HFD/STZ group there was only a significant increase in insulin from basal levels at 15 minutes (*P* < 0.05), and there was a much larger plasma glucose excursion (*P* < 0.001, compared to HFD/Veh controls). Consistent with these observations, levels of HbA1c (glycosylated haemoglobin) were significantly increased in the HFD/STZ group (10.5 ± 0.2%) compared to the HFD/Veh controls (7.4 ± 0.1%, *P* < 0.001). Alongside these metabolic changes, hindpaw mechanical withdrawal thresholds were significantly lowered in the HFD/STZ rats compared to the HFD/Veh rats (Figure 1(e)), indicative of the development of aberrant pain behaviour.

### 3.2. Decreases in Mechanical Withdrawal Thresholds in HFD/STZ Rats Are Prevented by the Antidiabetic Drugs Linagliptin and Metformin

**Figure 2:**
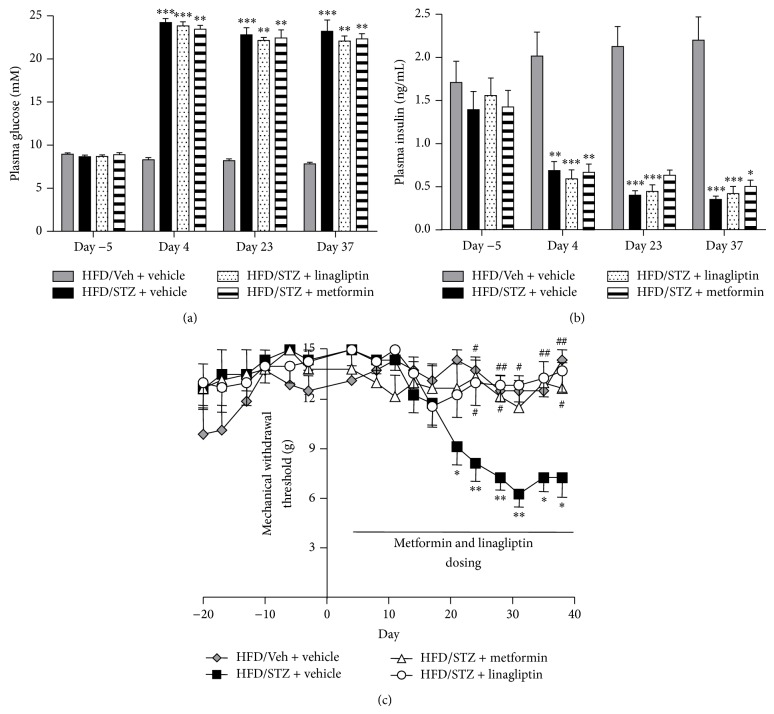
Effects of linagliptin and metformin on metabolic changes and mechanical withdrawal thresholds. Effects of linagliptin (3 mgkg^−1^, p.o.; *n* = 7) or metformin (200 mgkg^−1^, p.o.; *n* = 6) given daily from day 4 onwards on (a) fasting plasma glucose, (b) fasting plasma insulin, and (c) mechanical withdrawal thresholds in HFD/STZ rats, compared to the effects of vehicle in HFD/STZ rats (*n* = 8). Data from vehicle-treated HFD/Veh rats are also included for comparison as this group did not exhibit changes in mechanical withdrawal thresholds (*n* = 8). Linagliptin and metformin significantly prevented the decrease in hindpaw mechanical withdrawal thresholds in HFD/STZ rats compared to vehicle-treated HFD/STZ rats. Data are mean ± SEM. Analysis was by a Kruskal Wallis test with Dunn's post hoc test: ^*^ *P* < 0.05, ^**^ *P* < 0.01, and ^***^ *P* < 0.001 (compared to HFD/Veh); ^#^ *P* < 0.05, ^##^ *P* < 0.01 (compared to HFD/STZ).

Daily linagliptin (3 mgkg^−1^) and metformin (200 mgkg^−1^) treatment from day 4 after model induction did not significantly alter the changes in plasma glucose (Figure 2(a)) or insulin (Figure 2(b)) in the HFD/STZ rats. Despite the lack of effect of these interventions on the metabolic parameters, both linagliptin and metformin significantly prevented changes in the mechanical withdrawal thresholds in HFD/STZ rats (Figure 2(c)). The withdrawal thresholds of the linagliptin- and metformin-treated HFD/STZ rats were not significantly different from the HFD/Veh rats at any time point and were significantly different from the vehicle-treated HFD/STZ rats from day 24 until the end of the study.

### 3.3. Spinal Neuronal Response Properties in the HFD/STZ Model of Diabetes

**Figure 3:**
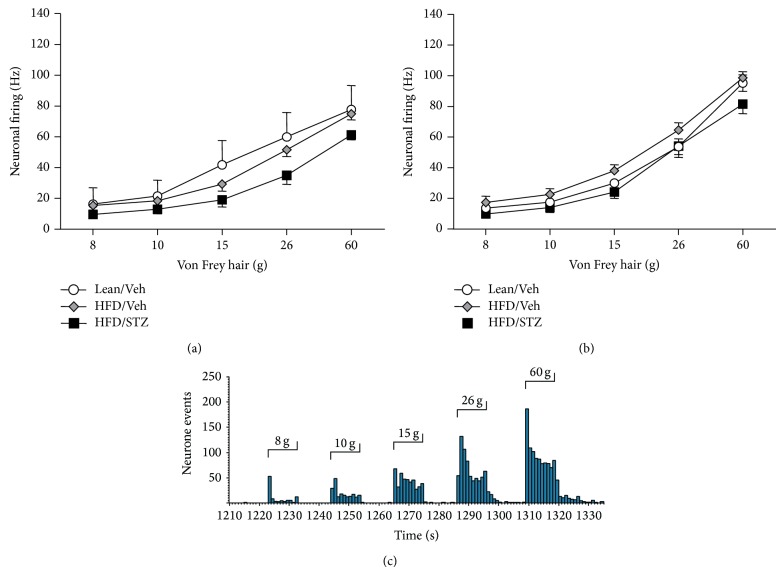
Effects of the HFD/STZ model on mechanically evoked responses of dorsal horn neurons. Comparison of the mechanically evoked responses of wide dynamic range dorsal horn neurones following stimulation of the receptive field on the hindpaw with a range of innocuous and noxious von Frey hairs in the Lean/Veh controls, HFD/Veh controls, and diabetic HFD/STZ rats at (a) day 49 and (b) day 78. Data are mean frequency of firing ± SEM, *n* = 6 rats per group, with an average of 15 neurones per group. (c) Representative trace of the evoked response of a single wide dynamic range dorsal horn neurone following mechanical (8, 10, 15, 26, and 60 g) stimulation of the hindpaw receptive field in the anaesthetised HFD/Veh rat.

To understand further the basis for the decreases in mechanical withdrawal thresholds in the HFD/STZ rats, the responses of neurones in the dorsal horn of the spinal cord were quantified. Electrophysiological recordings were performed at day 49 and day 78 following STZ. Wide dynamic range (WDR) dorsal horn neurones were characterized at day 49 (9 neurones in 6 Lean/Veh rats, 18 neurones in 6 HFD/Veh rats, and 18 neurones in 6 HFD/STZ rats). The range of the depths of dorsal horn neurones recorded was 480–1025 *μ*m, which corresponds to laminae V-VI. The vast majority of neurones recorded exhibited negligible or no spontaneous firing activity. Both the electrical and mechanically evoked responses of the WDR dorsal horn neurones were quantified. There were no significant differences in the electrical thresholds, latencies, or evoked responses of the WDR dorsal horn neurones in the HFD/STZ group compared to the Lean/Veh rats or the HFD/Veh rats (Table 1). Stimulation of the hindpaw receptive field of dorsal horn neurones with a range of von Frey hairs evoked a stimulus intensity-dependent increase in the firing of these neurones in all groups of rats (Figures 3(a) and 3(c)). There was a trend towards a decrease in the mechanically evoked responses of dorsal horn neurones in the HFD/STZ group for each weight studied, especially in the noxious range (15–60 g), although significance was not reached (Figure 3(a)). In a separate cohort of rats mechanically evoked responses of WDR neurones were characterised at day 78 following STZ (20 neurones in 6 Lean/Veh rats, 23 neurones in 6 HFD/Veh rats, and 17 neurones in 5 HFD/STZ rats). The range of the depths of dorsal horn neurones recorded was 685–948 *μ*m. No significant differences in the mechanical responses (Figure 3(b)) or the electrical thresholds, latencies, or evoked responses (data not shown) of the WDR dorsal horn neurones were observed in the HFD/STZ group compared to the Lean/Veh rats or the HFD/Veh rats.

**Figure 4:**
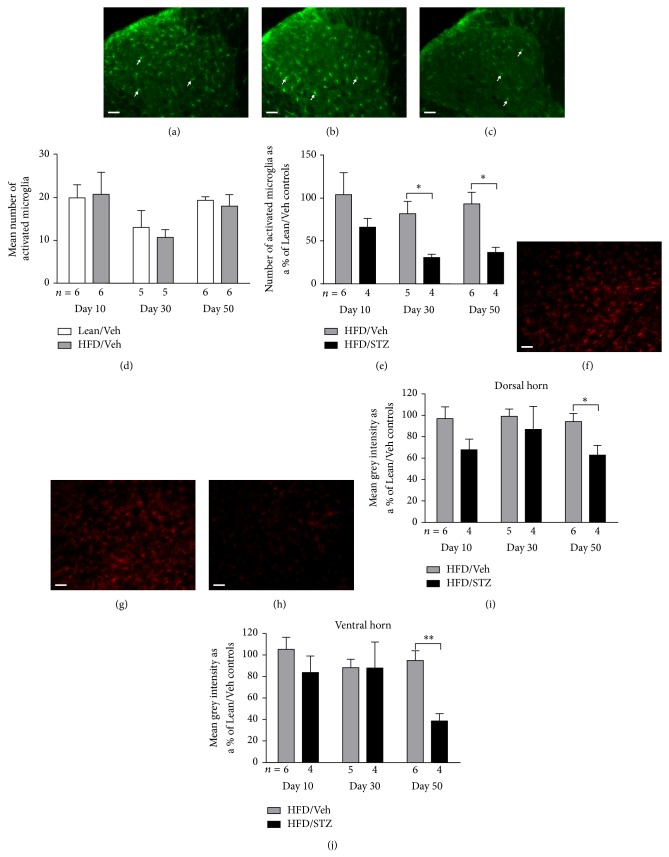
Effects of the HFD/STZ model on Iba-1 and GFAP staining in the spinal cord. ((a)–(c)) Representative images of Iba-1 staining in the dorsal horn of the spinal cord in a Lean/Veh, HFD/Veh, and HFD/STZ rat, from left to right. Scale bar: 50 *μ*m (20x magnification). Arrows indicate examples of Iba-1 positive cells morphologically identified as activated microglia. (d) Numbers of Iba-1 positive cells morphologically identified as activated microglia in the dorsal horn of the control groups (lean/vehicle and HFD/vehicle). (e) Effects of HFD/STZ on number of Iba-1 positive cells morphologically identified as activated microglia in the dorsal horn expressed as a percentage of the number in the Lean/Veh control group. The numbers of rats per group are shown below the *x*-axis, and 7 spinal cord sections per rat were processed. Data are mean ± SEM. Analysis was by a Mann-Whitney test: ^*^ *P* < 0.05. ((f)–(h)) Representative images of GFAP staining in the ventral horn of the spinal cord in a Lean/Veh, HFD/Veh, and HFD/STZ rat, from left to right. Scale bar: 50 *μ*m (20x magnification). Effects of HFD/STZ on mean grey intensity of spinal GFAP immunofluorescence in (i) the dorsal horn and (j) the ventral horn, expressed as a percentage of the mean grey intensity in the Lean/Veh control group. The number of rats per group is shown below the *x*-axis, and 7 spinal cord sections per rat were processed. Data are mean ± SEM, analysis was by a Mann-Whitney test: ^*^ *P* < 0.05, ^**^ *P* < 0.01.

To determine the potential contribution of changes in spinal immune cell activity to the aberrant pain behaviour observed in the HFD/STZ rats, the number of activated microglia in the dorsal horn of the spinal cord was quantified at different time points in the model. Figures 4(a)–4(c) are representative images of Iba-1 staining in dorsal horn spinal cord sections in a Lean/Veh, HFD/Veh and HFD/STZ rat, respectively. The average number of Iba-1 positive cells morphologically identified as activated microglia in the Lean/Veh control rats was comparable across the three time points (Figure 4(d)). The number of activated microglia in the dorsal horn of the spinal cord of the HFD/Veh control group was comparable to numbers in the Lean/Veh controls (Figure 4(d)). In the HFD/STZ group there was a significant decrease in the number of Iba-1 positive cells morphologically identified as activated microglia at days 30 and 50, compared to the HFD/Veh group (Figure 4(e)). To determine whether spinal levels of astrogliosis were also altered in the model, GFAP immunofluorescence in the dorsal and ventral horn of the spinal cord was quantified in Lean/Veh, HFD/Veh, and HFD/STZ groups (Figures 4(f)–4(h)). GFAP immunofluorescence was significantly decreased in the dorsal (Figure 4(i)) and ventral (Figure 4(j)) horn of the spinal cord in the HFD/STZ group at day 50, compared with the HFD/Veh group.

### 3.4. Effects of the PPAR*γ* Agonist, Pioglitazone, on Pain Behaviour in the HFD/STZ Model of Diabetes

**Figure 5:**
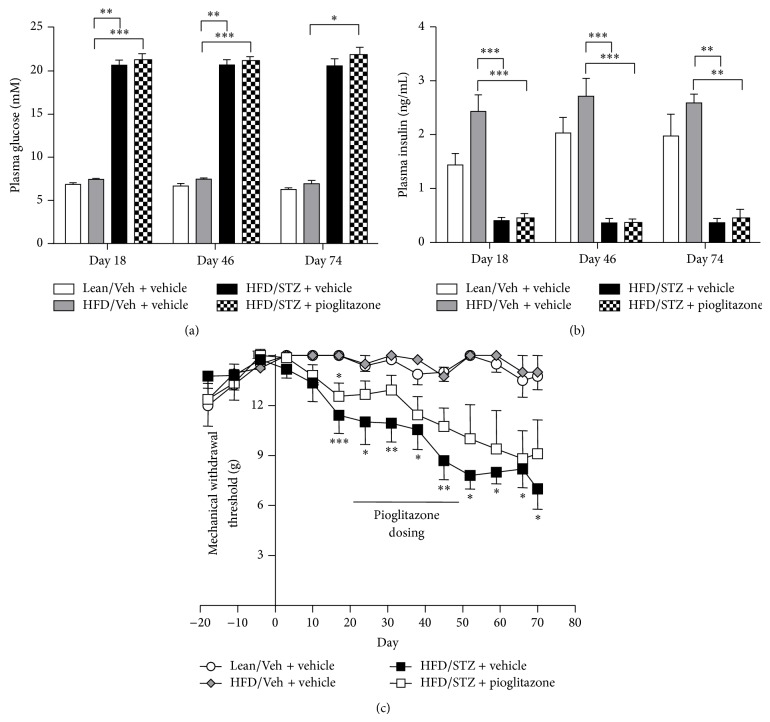
Effects of pioglitazone on metabolic changes and pain behaviour in HFD/STZ rats. Effects of pioglitazone treatment (10 mgkg^−1^, p.o.) versus vehicle from day 21–49, on (a) plasma glucose, (b) plasma insulin, and (c) hindpaw mechanical withdrawal thresholds in the HFD/STZ rat. Data are mean ± SEM. Group sizes day 0–49; Lean/Veh: *n* = 11; HFD/Veh: *n* = 11; HFD/STZ + vehicle: *n* = 10, HFD/STZ + pioglitazone: *n* = 10. Group sizes day 49 onwards; Lean/Veh: *n* = 6; HFD/Veh: *n* = 6; HFD/STZ + vehicle: *n* = 5; HFD/STZ + pioglitazone: *n* = 5. Analysis was by a Kruskal-Wallis test with Dunn's post hoc test: ^*^ *P* < 0.05, ^**^ *P* < 0.01, and ^***^ *P* < 0.001 (compared to HFD/Veh).

Previous studies have reported antinociceptive effects of the PPAR*γ* agonist pioglitazone in models of nerve injury induced neuropathy. Oral administration of pioglitazone (10 mgkg^−1^) or vehicle (1% methylcellulose) was carried out from day 21 after model induction for 28 days. Administration of pioglitazone did not alter the increase in plasma glucose (Figure 5(a)) or the decrease in plasma insulin (Figure 5(b)) induced by STZ treatment. Furthermore, although there was a separation in the hindpaw withdrawal thresholds of HFD/STZ rats treated with pioglitazone compared to vehicle-treated HFD/STZ rats (Figure 5(c)), there were no significant differences between the two groups over the period of treatment. On the final day of pioglitazone treatment (day 49) the mean mechanical withdrawal threshold for the pioglitazone-treated HFD/STZ group was 11 ± 1 g, compared to 9 ± 1 g for the vehicle-treated HFD/STZ group. Following cessation of pioglitazone treatment, hindpaw withdrawal thresholds continued to decline in the pioglitazone-treated HFD/STZ group to a comparable extent to that seen in the vehicle-treated HFD/STZ rats until the end of the study (day 78).

## 4. Discussion

In keeping with previous reports [12, 13], a high fat diet followed by a single dose of STZ resulted in increased plasma glucose and reduced plasma insulin secretion, coupled with a marked increase in water intake and HbA1c, characteristics associated with diabetes. In our study HFD/STZ rats exhibited weight loss and hyperglycaemia, which could be considered indicative of type 1* diabetes mellitus*; however, there was measurable and functional insulin present throughout the study duration, mimicking type 2 diabetes. In addition, the plasma glucose levels were consistent with those previously reported for this model [14], and glucose levels of* circa*. 23 mM have been previously reported in models of type 2 diabetes such as the ZDF rat [37]. A recent review has suggested that the HFD/STZ model might be a suitable model to study mechanisms associated with the final stages of type 2 diabetes [38]. Alongside the metabolic changes, HFD/STZ rats exhibited significant decreases in hindpaw mechanical withdrawal thresholds, a behavioural correlate of mechanical hypersensitivity, which was maintained for at least 120 days after STZ treatment. Decreases in hindpaw mechanical withdrawal thresholds in HFD/STZ treated rats were prevented by early intervention with two antidiabetic treatments (linagliptin and metformin) but not by the PPAR*γ* ligand, pioglitazone, in animals with well-established diabetes. Despite the HFD/STZ associated changes in pain behaviour, there was limited evidence for changes in sensory evoked responses of spinal neurones (at days 49 and 78). Spinal immune cell activity (microglia activation and astrogliosis) was not altered at an early time point in the model; however, at later time points levels of microglia activation and astrogliosis were significantly decreased.

In the present study we report a significant and sustained lowering of mechanical withdrawal thresholds from day 42 onwards in this model. The impact of the combination of a HFD/STZ treatment on the development of aberrant pain responses has only recently been reported, with mixed effects described, which may arise due to differences in the dose of STZ and the duration of study. Combination of an HFD with a lower dose of STZ (35 mgkg^−1^) in the rat produced milder changes in blood glucose concentrations and greater gains in body mass and was not associated with changes in thermal or mechanical sensitivity [39], supporting the notion that the extent of the metabolic changes may be pivotal to the development of pain behaviour in this model. By contrast, hypersensitivity (at day 15), followed by a decline in sensitivity, to cold and thermal stimuli has been reported in another study of this model [18]. In addition, there was an increase in response frequency to a noxious mechanical stimulus, which also peaked at day 15 and then declined over time, but only transient small effects on mechanical withdrawal thresholds at 30 days after model induction in the rat [18]. Collectively these studies are broadly supportive of this model leading to changes in pain responses that are clinically relevant. However, alterations in mechanical sensitivity are only robust at later stages of the model, highlighting the important relationship between the temporal development of the metabolic changes and the progression of pain behaviour. Consistent with the published literature [40], our data suggest that slower and longer-term cellular and molecular changes that are brought into play by elevated blood glucose and/or decreased insulin underlie the decreases in hindpaw withdrawal thresholds. These changes, such as the increase in oxidative stress, lead to a whole raft of changes, which can cause damage to neurones and can result in an increase in sodium channels and ectopic discharges, altering sensory afferent function [41, 42]. Although not determined in the present study, the consumption of high fat diets and the depletion of insulin are widely reported to lead to elevated levels of plasma-free fatty acids (FFAs; [43]) such that HFD/STZ animals may exhibit high levels of FFAs [44, 45]. Interestingly, such increases in plasma FFAs have been linked to changes in peripheral nerve function [46] and microglia inflammatory responses [47]. Accordingly, it is feasible that increases in plasma FFAs in the HFD/STZ-treated rats contributed to the changes in hindpaw withdrawal thresholds reported herein. Indeed, if true, it may be that the action of linagliptin and metformin to improve diabetic neuropathy is related, at least in part, to an action of the drugs to control plasma lipids. Such a hypothesis is consistent with studies that have demonstrated that DPP-4 inhibitors and metformin improve blood lipid profiles in both preclinical and clinical studies [48–50].

To understand the potential contribution of changes in spinal excitability to the manifestation of aberrant pain behaviour, sensory afferent evoked responses of spinal neurones were recorded. Our observation of a nonsignificant decrease in evoked responses of spinal neurones at day 49 in HFD/STZ treated rats is consistent with reported decreases in evoked spinal neuronal responses in another model of diabetes [7], as well as a surgical model of neuropathy [51], and the cisplatin-induced chemoneuropathy model [52], which may reflect deficits in sensory inputs at this stage. By contrast, at the later time point the magnitude of evoked firing of the WDR neurones was comparable between HFD/STZ and HFD/vehicle treated rats suggesting that local or supraspinal [53] compensatory mechanisms may override the sensory afferent deficits observed at earlier time points. Overall, our electrophysiological studies reveal evidence of subtle time-dependent changes in spinal neuronal excitability in this model, but these do not parallel the overt pain behaviour. The lack of concordance between the magnitude of the evoked responses of spinal neurones and the behavioural manifestation of mechanical allodynia (measured as a lowering of hindpaw withdrawal thresholds) is not unique to the study of models of diabetic neuropathy (see, e.g., [54]). Indeed, these differences in the neuronal and behavioural responses point to potential roles of higher brain centres in driving hyperexcitability in these models of chronic pain [55].

The contribution of activated microglia and astrocytes to mechanical hypersensitivity has been widely studied in models of surgically induced neuropathic pain where it is generally accepted that both types of immune cell play an important role in the development and/or maintenance of aberrant pain responses [56]. Numbers of activated microglia in the spinal cord were unaltered at day 10 in the HFD/STZ group, consistent with data from the db/db mouse model of type 2 diabetes [57]. By contrast, numbers of spinal microglia were increased at early time points (2 weeks) in the STZ model [10]. At the later time point (day 50) in our study, the numbers of activated microglia in the spinal cord of HFD/STZ rats were significantly decreased compared to controls, suggesting that if changes in microglia activation in the spinal cord contribute to the associated changes in pain behaviour in this model, these are likely to be transient in nature as is the case for other models of chronic pain. It is noteworthy that a higher dose of STZ (60 mg/kg) resulted in a significant increase in numbers of activated microglia in the spinal cord up to 6 months following model induction [58], illustrating marked differences between the models in this regard. Consistent with previous reports in models of diabetic neuropathy [11, 12, 36, 59], spinal GFAP immunofluorescence was significantly decreased in both the dorsal and the ventral horns of the spinal cord by day 50 in the HFD/STZ model. Indeed, previous studies have reported a link between diabetes and GFAP expression in the brain (see references in [59]). A likely consequence of a downregulation in astrocyte number in the spinal cord is an increase in spinal levels of glutamate and associated excitotoxicity [60]. Although the causal link between diabetes and changes in astrocyte function is yet to be fully elucidated, insulin is known to regulate both the differentiation and function of astrocytes [61]. Exogenous insulin treatment can prevent the diabetes induced changes in astrocyte glutamate uptake and GFAP expression in the brain [59]. Interestingly, circulating levels of insulin are correlated with allodynia [62] and insulin treatment can also prevent or reverse mechanical allodynia [63].

Treatment with linagliptin and metformin, 4 days after injection with STZ, prevented the development of mechanical hypersensitivity in the HFD/STZ model. The effects of linagliptin on pain behaviour in the HFD/STZ model reported herein are consistent with previous reports that DPP-4 inhibitors can improve thermal nociception and reduce mechanical hypersensitivity in the STZ model, without affecting glucose and insulin levels [64]. These effects may reflect a peripheral site of action, as improvements in sensory thresholds and nerve fibre loss have been reported [65]. In our study, the effects of linagliptin and metformin on pain behaviour were independent of changes in glycemic control, as neither drug altered the HFD/STZ induced changes in plasma glucose and insulin, or body weight, pointing to alternative mechanisms of action. Putative mechanisms include the effects of linagliptin arising from inhibition of DPP-4 and the resultant beneficial effects of increasing plasma levels of GLP-1 on nerve function [37], supporting a mechanism independent of glycemic control [66]. It is noteworthy that increased *β*-cell mass and small but significant improvements in an OGTT in the HFD/STZ mouse model following dosing with the DPP4 inhibitors, saxagliptin, or sitagliptin, either before, or shortly after STZ administration have also been reported [67]. The ability of the AMPK activator metformin to inhibit pain behaviour in the HFD/STZ model in the absence of overt changes in plasma insulin and glucose is consistent with reports that metformin, and other AMPK activators attenuate pain responses in other rodent models of neuropathic [29, 68] and inflammatory pain [69]. Activation of AMPK inhibits mTOR and ERK signalling pathways and insulin receptor substrate- (IRS-) mediated feedback signalling, which has multiple downstream effects including inhibition of local protein synthesis [70] and decreased phosphorylation of the Na_v_1.7 channel [29, 71]. Metformin has also been shown to increase levels of GLP-1 in mice, rats, and humans, suggesting that it may also share a common mechanism with linagliptin [72].

The last part of this study investigated whether the thiazolidinedione pioglitazone, which can prevent the development of mechanical allodynia and hyperalgesia in nerve-injury induced models [73], can alter established pain behaviour in HFD/STZ rats. Four-week treatment with pioglitazone once pain behaviour was established did not alter the decreases in hindpaw mechanical withdrawal thresholds, compared to the vehicle-treated HFD/STZ rats. Higher doses of pioglitazone (30–100 mgkg^−1^) have been shown to reverse established mechanical hypersensitivity 2 weeks after the induction of a surgical model of neuropathy [32], although selectivity at these doses may be an issue. Consistent with previous reports [74], pioglitazone treatment did not alter glucose or insulin levels in the HFD/STZ rats, probably reflecting the low levels of fasting plasma insulin (sixfold lower than HFD/Veh controls) in the HFD/STZ model which indicate that rats are insulin deficient, rather than insulin resistant, limiting the extent of any improvement in peripheral insulin sensitivity. These low levels of insulin may also help to explain why linagliptin and metformin had no effect on glucose or insulin levels, as the rats were unable to secrete any more insulin.

## 5. Conclusions

We have characterised the temporal development of alterations in mechanical withdrawal thresholds, a clinical relevant pain endpoint, in the HFD/STZ model of diabetes and demonstrated that the currently prescribed antidiabetic drugs linagliptin and metformin prevent the development of this pain behaviour. Despite the reported ability of preventative/early treatment with pioglitazone to attenuate the development of aberrant pain responses in models of neuropathy, this intervention did not alter established pain behaviour in the HFD/STZ model of diabetic neuropathy. Future mechanistic studies in this model have the potential to advance our understanding of the biological processes that lead to the manifestation of aberrant pain associated with peripheral diabetic neuropathy and provide a platform for the exploration of new analgesic approaches.

## Figures and Tables

**Table 1:** Effects of the HFD/STZ model on electrically evoked responses of dorsal horn neurones.

	Lean/Veh	HFD/Veh	HFD/STZ
C-fibre threshold (mV)	1.5 ± 0.2	1.3 ± 0.1	1.2 ± 0.1
Latency (ms)	228 ± 13	189 ± 11	192 ± 19
A*β* (total APs)	138 ± 12	139 ± 8	111 ± 8
A*δ* (total APs)	161 ± 24	123 ± 19	105 ± 21
C (total APs)	392 ± 67	251 ± 37	276 ± 48
Postdischarge (total APs)	440 ± 98	309 ± 48	402 ± 82

Comparison of thresholds for electrical activation of C-fibres, latencies of C-fibre evoked responses of WDR dorsal horn neurones, and electrically evoked responses of WDR neurones in the HFD/STZ model of diabetes, compared to HFD/Veh and Lean/Veh controls at day 49 after model induction (*n* = 6 neurones in *n* = 6 rats per group). Data are mean ± SEM. There were no statistical differences between the groups (Kruskal Wallis test).
